# Evaluation of the spatial distribution of scenic resources based on 3S technology: A case study of the Yesanpo National Park

**DOI:** 10.1371/journal.pone.0269841

**Published:** 2022-07-08

**Authors:** Zhe Jia, Anchen Qin

**Affiliations:** College of Landscape Architecture and Tourism, Hebei Agricultural University, Baoding, China; Đại Học Duy Tân: Dai Hoc Duy Tan, VIET NAM

## Abstract

Evaluation of scenic resources is imperative in national park planning, and 3S technology has been applied for this purpose because it effectively leverages modern geo-informatics technology. We investigated the scenic resources in the Yesanpo National Park based on 3S technology. The Nearest-Neighbor Index, Kernel density estimation, imbalance index, and geographical concentration index in modern geography were introduced into the spatial distribution evaluation of scenic resources, and here, for the first time, the spatial combination index of scenic resources was proposed as one of the quantitative analysis indices of spatial distribution of scenic resources. Moreover, the spatial combination distribution characteristics of scenic resources were analyzed quantitatively and graphically. The characteristics of the spatial distribution of scenic resources in the Yesanpo National Park were as follows: The spatial distribution type of scenic spots in the Yesanpo National Park is a clustered type. The spatial distribution of the scenic resource groups in the Yesanpo National Park was extremely imbalanced and highly concentrated. Based on the identification of scenic resources, we evaluated the scenic resources of each area and propose sustainable development plans for each area. This evaluation method can be applied to similar national parks to promote the quantitative and graphical evaluation of the spatial distribution of scenic resources and provide support for the decision-making processes related to national park planning and management.

## Introduction

In the 1980s, national parks, such as Huangshan and Yesanpo, began to develop tourism—the Yesanpo National Park is a typical representative of tourism development in northern China. Since its official opening for tourism in 1986, the economic development of the national park and surrounding areas have been promoted, and Yesanpo has been awarded the “China Environmental Protection Ecological Demonstration Zone, National Agricultural Tourism Demonstration Site, and National Cultural Industry Demonstration Base, 2018 Chinese brand tourist attractions TOP20” and many other awards. In recent years, the coordinated development of tourism in the Yesanpo National Park has received a great deal of attention. The national park is located in the Yanshan-Taihang Mountain contiguous and extremely poor area. However, these impoverished areas have rich and diverse natural scenic resources and great potential for tourism development. The national park actively explores the tourism poverty alleviation idea of “national parks drive village development,” and regards tourism as an important measure to promote the development of poverty-stricken areas. It has been selected in the “World Tourism Alliance Best Practice in Poverty Alleviation through Tourism,” which is a typical area for tourism to promote poverty alleviation and rural revitalization. With the rapid development of the tourism industry, understanding the spatial evaluation of scenic resources has also developed. How to assess the spatial distribution of scenic resources scientifically and accurately is helpful to select suitable areas to develop tourism products, formulate reasonable and effective national park planning, and promote the sustainable use of scenic resources, which is an important basis for the sustainable development of national parks.

The spatial evaluation of scenic resources needs scientific and reliable technical support. The advantages of evaluating the spatial distribution of scenic resources based on 3S technology are apparent and the application prospects are broad. Furthermore, 3S technology includes Remote Sensing (RS), Geography Information System (GIS), and Global Positioning System (GPS) [1]. In recent years, owing to its high efficiency and convenient data processing advantages, it has been applied to the collection and positioning of spatial information of scenic resources, identification and evaluation, analysis and mapping. In particular, it has evident advantages in the investigation and evaluation of natural resources in national parks, and the spatial evaluation accuracy of scenic resources, such as landforms and vegetation, is relatively high [2–6].

1. When identifying and evaluating scenic resources, RS technology is less affected by terrain, traffic, weather as well as other conditions, while it has wider identifying and evaluation range than traditional visual observation; therefore, it can comprehensively evaluate scenic resources in national parks. High-resolution RS images, such as GF-2, have the advantages of rich information, high definition, intuitive images, strong real-time performance, fast image formation, and fast data acquisition, which greatly reduce the difficulty of scenic resource evaluation and have apparent advantages in resource evaluation.
2. The use of GIS technology to establish a scenic resource database can be used for digitization, standardization input, and storage of various data, such as scenic resource spatial data, texts, and charts. It can also perform operations such as spatial analysis, data supplementation, and update, and result output on scenic resource data, which can improve the efficiency of the use and management of scenic resource data and improve the systematic and scientific nature of spatial evaluation.
3. The use of GPS technology can provide accurate spatial positioning information for the evaluation of scenic resources. It has the advantages of fast positioning, high accuracy, and all-weather operations, and can help to verify spatial evaluation results.

Therefore, it is urgent to study scientific and operational evaluation of the spatial distribution of scenic resources based on 3S technology. However, to date, in-depth and systematic research on the evaluation of the spatial distribution of scenic resources based on 3S technology has not been conducted in academia.

Scenic spots specifically refer to independent scenic resources developed for tourism and have independent viewing or utilization value. Research on the spatial structure of scenic spots in academia started relatively late, mostly focusing on empirical studies of tourist attractions. Research on the spatial structure of tourist attractions can be summarized as follows: the spatial structure of tourist attractions on different scales (macro: country, region; mesoscale: province, city; micro: a single scenic spot); the influencing factors of the spatial structure of tourist attractions; and the analysis methods of the spatial structure of tourist attractions [7, 8].

The systematic study of the spatial structure of tourist attractions began in the 1960s [7]. Deasy and Griess [9] used tourism indifference curves, tourism cost lines, and other methods to analyze the competition between similar tourist attractions in Pennsylvania, USA. Wilson [10] compared the principle of random model variables by combining research on the spatial structure of the scenic spot and constructed a random variable gravitational model of the scenic spot. Before the 1990s, scholars mostly used mathematics or basic models from other disciplines to study the spatial structure of scenic spots.

After the 1990s, research on the spatial structure of tourist attractions matured. Through the study of literature, the following characteristics were found.

1. In terms of research theory, it focuses on related disciplines, such as urban geography, economic geography, and tourism geography [11–13], and analyzes the spatial structure of scenic spots through improved spatial structure methods and theoretical models.
2. In terms of research objects, the focus is on the planar spatial structure of a single tourist attraction, multiple tourist attractions, or scenic urban spots. In recent years, large-scale research has gradually decreased, and meso-microscale research has continued to increase [14]. However, there are very few related studies on the three-dimensional spatial structure of national parks.
3. In terms of research content, it mainly focuses on the spatial structure distribution characteristics, spatial mutual relations, spatial influencing factors, spatial structure evolution, and spatial structure optimization of the scenic area [15, 16].
4. As a research method, the nearest-neighbor ratio R, connectivity index (α, β, γ index), geographic concentration index, accessibility index, average path length, compactness index, fractal theory, and network analysis method have been applied [17–21]. In recent years, with the widespread application of GIS spatial analysis methods, GIS has also been introduced into the study of the spatial structure of scenic areas. It can be analyzed graphically and digitally to determine the spatial structure of a scenic area more intuitively. However, structural analysis needs to be further strengthened [22–24].

In research related to the spatial identification and evaluation of scenic resources, previous studies mostly focused on individual analysis and evaluation, and rarely on the identification and evaluation of scenic resources from the perspective of spatial combination and structure. However, in actual planning, spatial combinations are more important. Effective areas exhibiting a better spatial combination are often areas where scenic resources are concentrated, and they are also key areas for national park development and planning [20]. Therefore, here, we introduce the mathematical models used to evaluate the distribution of spatial elements in modern geography, such as the geographical concentration index, and other quantitative analyses, to evaluate the spatial combination of scenic resources and their distribution characteristics.

This study applies the GIS spatial analysis method to the spatial combination and distribution analysis of the Yesanpo National Park, and combines the requirements for the sustainable development of national parks. The “spatial combination index of scenic resources (SCISR)” is proposed for the first time. Taking the Yesanpo National Park as an example, combined with the thematic map of the spatial distribution of scenic resources, we analyze the scenic resource groups and their distribution characteristics from the perspective of three-dimensional space. The results are verified with field survey data in the study area, and the method is proven to be scientific and feasible. The method provides an objective, accurate, and visual way for national park managers and planning units to analyze the combination of scenic national park resources and the characteristics of their distribution from a spatial perspective. The evaluation system helps to improve the science and rationality of the evaluation of the spatial distribution of scenic resources. It is also helpful for identifying areas with relatively concentrated scenic resources and high development potential, and providing a theoretical basis and technical support for the spatial combination and structural analysis of national parks, as well as data support for sustainable development.

## Materials and methods

### Study area

The total area of the Yesanpo National Park is 505.48 km^2^. It is located in the northwest of Laishui County, Baoding City, Hebei Province, China, in the deep mountain area at the junction of Taihang Mountain and Yanshan Mountain. The Yesanpo National Park is spread over a large area with a complex terrain, diverse types of scenic resources [25], and a variety of landscapes, such as mountains, clear waters, and strange gorges. The resources and geological relics are typical and unique, forming unique erosion narrow gorges, granite structural waterfalls, canyons, and karst cave spring landscapes. It is a global geological park, a national park in China, and a national eco-tourism demonstration area [26, 27]. It was officially opened in 1986 to attract tourists. It was approved as a national park in China in 1988, and has strong representation in this area. There are six scenery areas in the area: Baili Gorge, Longmen Tianguan, Yugu Cave, Juma River, Jinhua Mountain, and Baicao Pan.

### Data and preprocessing

**Fig 1:**
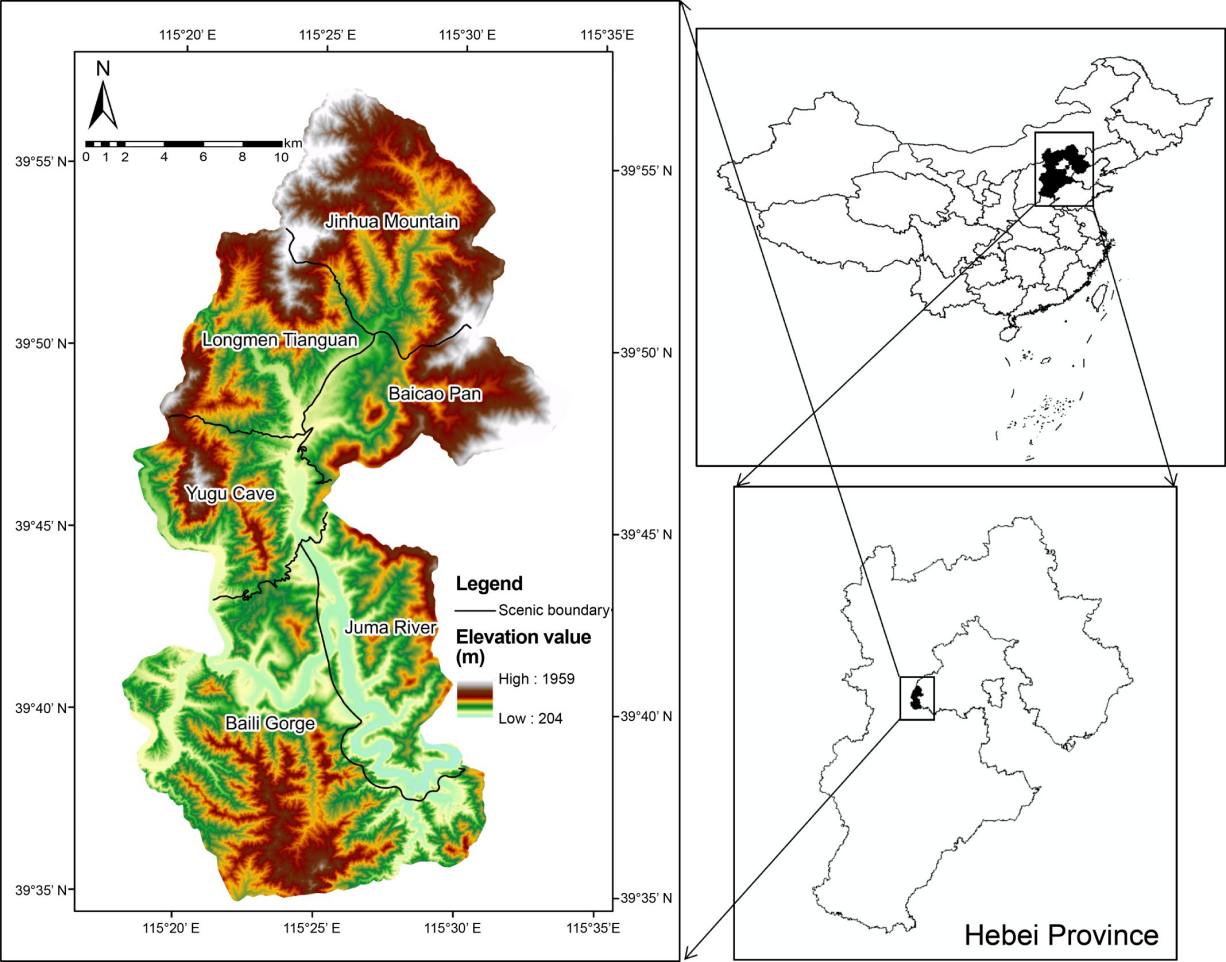
Remote sensing image of the Yesanpo National Park. (Republished from [https://directory.eoportal.org/web/eoportal/satellite-missions/g/gaofen-2] under a CC BY license, with permission from [Hebei Zhongke Sino Star Information Technology Co., Ltd], original copyright [2018]).

According to research needs, the following data and information were used:

Imaging data of Gaofen-2 (GF-2) of Yesanpo National Park obtained on April 16, 2018. The multi-spectral resolution of GF-2 is 3.2 m [28], and the panchromatic resolution can reach 0.8 m [29]. ENVI (Exelis Visual Information Solutions, American) and ArcGIS (Esri, American) were used to perform preprocessing, such as orthorectification, fusion processing, geometric precision correction, and mosaic cropping of GF-2 image data (Fig 1).

GPS was used to investigate the coordinates of 135 scenic spots available and the ones under planning, and these were recorded in the GIS database, as shown in Table 1.

**Table 1:** List of scenic spots at the Yesanpo National Park.

Scenery area	Scenic spot
**Baili Gorge (51)**	1 Tian Ditch, 2 Optimus Stone Pillars, 3 Strange Peaks, 4 Echo Walls, 5 Cross-Bedding, 6 Plate Mountains, 7 Narrow gorge formation, 8 No Sky, 9 Yixin Waterfall, 10 Wu Mishan Group, 11 Connecting Heart Bridge, 12 stacked waterfall cave sky, 13 Shuilian cave, 14 Luofeng slope, 15 wave marks, 16 collapse and accumulation, 17 golden rooster report, 18 modern calcification, 19 Niujiao Peak, 20 turtle exploration, 21 Chenniu Lake, 22 One-line sky, 23 python out of the cave, 24 tiger’s mouth, 25 winding path, 26 natural bridge, 27 Wuzi Dengke, 28 gold thread hanging needle, 29 looking back Guanyin, 30 Huarong Road, 31 Shuangxin Waterfall, 32 Tiger Spring, 33 Qingxin Waterfall, 34 Spiral bedding, 35 Wangjing Tuo, 36 Golden Autumn Flame Mountain, 37 Emerald Valley, 38 Apricot Hill, 39 Southwest Terrace, 40 Daqing Cliff, 41 Iron Lock Cliff, 42 Longtan Yingyue, 43 Xianguan Guiding Road, 44 Squirrel’s Fengshou, 45 Han Dynasty Castle, 46 Yesanpo Geology, 47 Huoxiu Theater, 48 Hebei Agricultural and Sideline Products Exhibition Center, 49 Haitang Ferry, 50 Lingzhi square, 51 Liujiahe Revolutionary Martyrs Cemetery
**Longmen Tianguan (28)**	1 Tianshi Lecture, 2 Heavenly Dogs Serve the Moon, 3 Fairy World Waterfall, 4 Ganlu Waterfall, 5 Tianti Waterfall, 6 Feilong Waterfall, 7 Basalt, 8 Jushen Waterfall, 9 Lotus Waterfall, 10 God Lion Waterfall, 11 Yinhe Waterfall, 12 Fish Scales Stone, 13 Wangcheng Terrace, 14 Qiancheng Rock, 15 Longmen Temple, 16 Cultural Corridor, 17 Vortex Stone, 18 Cliff Rock Carving1, 19 Sage’s Ferry, 20 Rolling Wood Thunder Rock, 21 Cliff Rock Carving2, 22 Longmen Ferry, 23 Dalongmen Castle, 24 Beizhuang Site, 25 Fenghuo Vision, 26 Nanhu Qianti, 27 Thousand Year Ginkgo, 28 Caishuan Great Wall
**Yugu Cave (6)**	1 Buddha Cave Tower, 2 Egg Tuo, 3 Yunling Sunset, 4 Yugu Spring, 5 Yugu Cave, 6 Shanxiang Tea Garden
**Baicao Pan (29)**	1 Former residence of the old man official, 2 Daze Reservoir, 3 Baicaotang, 4 Yeshanju, 5 Beibianqiao Homo sapiens fossil site, 6 Ancient Chinese tree, 7 Bianqiao distant mountains, 8 Juyunfeng, 9 Observatory, 10 Alpine meadows, 11 Baicao Pan Main Peak, 12 Five Finger Peaks, 13 Shoushan Weng, 14 Bei Shan Weng, 15 Wild Walnut Forest, 16 Wind Stone, 17 Forest Sea, 18 Qingliang Valley, 19 Small Glaciers, 20 Twelve Pines, 21 Oak Forest, 22 Wannian spring, 23 Qingliang Pavilion, 24 Yiyou Pavilion, 25 Rhododendron, 26 Birch Forest, 27 Dingxiangyu, 28 Dishui Cliff, 29 Great Glacier
**Jinhua Mountain (11)**	1 Old Pines, 2 Lingnantai Ancient Village of Ming and Qing Dynasties, 3 Inviting Star Terrace, 4 Yuluobitao, 5 Cypress House, 6 Sunset Peak, 7 Xieshan Pavilion, 8 Hanging Temple, 9 Qingquan Temple, 10 Zhuangli Reservoir, 11 Wuzhishan
**Juma River (10)**	1 Linping lansheng, 2 Cuiping Shazhu, 3 Fangxin Manyuan, 4 Huahaidu, 5 Sand Mang Block Road, 6 Ancient cypress, 7 Lingshui Dock, 8 Shangzhuangdu, 9 Xiangan Yuejing, 10 Dietai Wangfeng

The DEM (Digital Elevation Model) data of the Yesanpo National Park, with a resolution of 12.5 m (NASA official website ALOS DEM data).

Survey data of forest resource planning and design in the Yesanpo National Park in 2018.

### Spatial distribution evaluation index

The spatial distribution type (random type, uniform type, and cluster type distribution) of the unit of scenic resources (scenic spots) is determined by the Nearest-Neighbor Index (NNI) [30]. The Kernel density estimation (KDE) method is used to determine the spatial aggregation area of the unit of scenic resource (scenic spots), and analyze the distribution density of the unit of scenic resource (scenic spots) in the surrounding area per unit area. We use the imbalance index to analyze the equilibrium degree of scenic resource groups in space (balanced, unbalanced), the geographic concentration index to analyze the concentration degree of scenic resource groups in spatial distribution, and the SCISR to quantitatively evaluate the value of the spatial distribution of scenic resource groups. Through the above indices, we provide a basis for the scientific integration and development of national parks, as shown in Table 2.

**Table 2:** Spatial distribution evaluation index.

Spatial distribution evaluation index	Formula	Description	Significance
Nearest-Neighbor Index (NNI)	$R=\frac{r1}{rE}$ (1)	*r*_*1*_ represents the average value of the actual distance between each point and its nearest neighbor. *r*_*E*_ represents the theoretical nearest-neighbor distance. *n* represents the number of scenic spots. *A* represents the area of the national park. *D* represents the point density.	NNI is used to measure the mutual proximity of scenic spots in the spatial distribution [31]. In this study, the NNI is used to determine the spatial distribution types of scenic spots, which are divided into random, uniform, and cluster.
	$rE=\frac{1}{2\sqrt{n/A}}=\frac{1}{2\sqrt{D}}$ (2)		When R = 1, the scenic spots are randomly distributed. When R>1, the scenic spots are uniformly distributed. When R<1, the scenic spot distribution is clustered. The smaller the R, the higher the concentration of scenic spots [32]. The smaller the *P*-test and *Z*-test values, the more the spatial distribution tends to be clustered.
Kernel Density Estimation (KDE)	$fn(x)=\frac{1}{nh}\sum_{i=1}^{n}k(\frac{x-Xi}{h})$ (3)	$k(\frac{x-Xi}{h})$ represents the kernel function, *h* represents the bandwidth, *n* represents the number of scenic spots, (*x−X*_*i*_) represents the distance from the estimated scenic spots *x* to *X*_*i*_.	KDE is used to quantitatively analyze the density of scenic spots using a moving cell [33, 34]. The assumption is that geographic events can occur at any location in space. The probability of event occurrence is high in areas where scenic spots are dense and low in areas where scenic spots are sparse. KDE is used to determine the center position of the scenic spots. The density is highest in the center and decays with distance. Finally, the density is zero at the extreme distance. The denser the distribution of the elements, the higher the probability of a geographic event (e.g., tourism behavior) in this area.
Imbalance Index	$S=\frac{\sum_{i=1}^{n}Yi-50(n+1)}{100n-50(n+1)}$ (4)	*S* represents the imbalance index, *n* is the total number of scenic resource group types, and *Y*_*i*_ represents the proportion of the number of graphic blocks of each scenic resource group type among the total number of scenic resource group types in the national park, and the accumulation of the i-th place after sorting from the largest to the smallest percentage.	Imbalance Index is used to quantitatively analyze the degree of imbalance in the spatial distribution of scenic resource groups [35].
			*S* is between 0 and 1. When *S* = 0, all scenic resource groups are distributed in the national park absolutely and evenly; when *S* = 1, all scenic resource groups are concentrated in a certain area of the national park.
Geographic Concentration Index	$G=100\times \sqrt{\sum_{i=1}^{n}{\left(\frac{Xi}{T}\right)}^{2}}$ (5)	*T* represents the total number of graphic blocks of scenic resource groups, *X*_*i*_ represents the number of graphic blocks of the i-th scenic resource group, *n* represents the total number of scenic resource group types.	The Geographic Concentration Index is used to quantitatively measure the spatial distribution and concentration of scenic resource groups. *G* had a value between 0 and 100 [36, 37]. The closer *G* was to 0, the more scattered the spatial distribution of scenic resources, and the closer *G* was to 100, the more concentrated the spatial distribution of scenic resource groups. *Ḡ* represents the geographical concentration index when the scenic resource groups are evenly distributed in the national park. If *G*>Ḡ, it means that the scenic resource groups are concentrated; conversely, *G*<Ḡ means that the scenic resource groups are distributed.

### Spatial combination index of scenic resources (SCISR)

To evaluate the viewing or utilization value of the spatial distribution of scenic resource groups more scientifically and to objectively, and provide references for national park planning staff and relevant government departments, we proposed the SCISR indicator, which indicated the viewing or utilization value of scenic resource groups in a certain area. The calculation process was as follows:

1. First, the expert scoring method was used to assign values to each scenic resource and its combination type, and then the corresponding score for each scenic resource and its combination type was recorded in the scenic resource GIS database.
  To improve accuracy, we used the coefficient of variation to analyze the degree of consistency of the expert scoring using the following expression [37]:
$$Mj=\frac{\sum_{i=1}^{n}Xij}{n},Sj=\sqrt{\frac{\sum_{i=1}^{n}{\left(Xij-Mij\right)}^{2}}{n-1}},Vj=\frac{Sj}{Mj}$$ (6)
  where *V*_*j*_ refers to the coefficient of variation of the j-th index, which represents the degree of coordination. The lower the V value, the more consistent the experts’ scores. *M*_*j*_ is the average value of the j-th index. The higher the M value, the more satisfied the experts are with the index. *S*_*j*_ represents the standard deviation of j-th index, *X*_*ij*_ represents the score value of the i-th expert for the j-th index, and n represents the number of members of the expert group.
2. ArcGIS’s neighborhood analysis tool was used to determine the minimum analysis area, calculate the evaluation value of each neighborhood scenic resource group in the analysis area, assign the evaluation value to each pixel in the corresponding analysis area and calculate the scores of the scenic resources and their combinations.
3. Vector images of scenic resource spatial combination index were generated and converted to raster images. SCISR maps were established and the scenic resource groups were quantitatively and graphically evaluated. The value of each grid in the index map was the SCISR in the area, and the higher the value, the higher the viewing or utilization value of the scenic resource groups.

### Identification and evaluation process

First, the object-oriented method of eCognition (Definiens Imaging, Germany) was used to identify the scenic resource data of Yesanpo GF-2 imagery [38]; thereafter, it was linked to the spatial and characteristic data obtained from DEM data. This was followed by the correction of survey data of forest resource planning and design to establish a GIS database. Subsequently, it was classified according to the spatial structure characteristics of the scenic resources. According to the structural characteristics of scenic resources in a three-dimensional space, it is divided into two spatial structure layers: geomorphological layer and surface cover layer. The surface cover layer is overlaid on the geomorphological layer. The scenic resources of the two spatial structure layers are superimposed and combined to form a variety of scenic resources groups [39] with clear semantics and rules [40, 41], as shown in Table 3.

**Table 3:** Types of scenic resources.

	Geomorphological layer (8)	Surface cover layer (10)
Small category	Plain, Platform, Hill, Low-relief mountain, Intermediate-relief mountain, High-relief mountain, Canyon/river valley, Peak	River, Lake, Deciduous broad-leaved forest, Coniferous forest, Deciduous shrub, Grassland, Farmland, Gardens, Swamp, Rural settlements

NNI, KDE, imbalance index, geographic concentration index, and SCISR were used to analyze and evaluate the spatial distribution characteristics and developmental value of scenic resources.

## Results

### Construction of the scenic resource model

**Fig 2:**
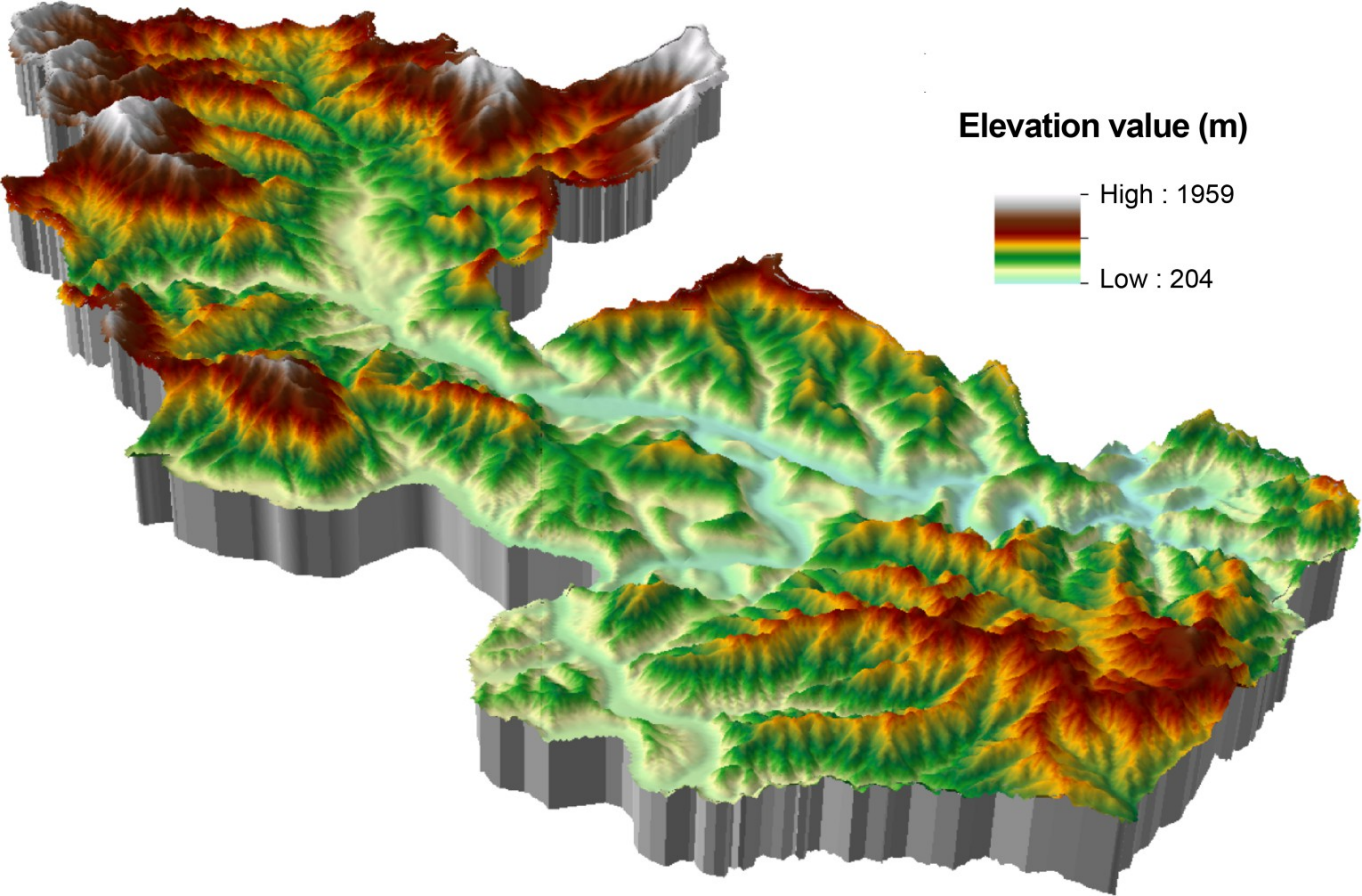
3D terrain model of the Yesanpo National Park. (Republished from [https://directory.eoportal.org/web/eoportal/satellite-missions/g/gaofen-2] under a CC BY license, with permission from [Hebei Zhongke Sino Star Information Technology Co., Ltd], original copyright [2018]).

**Fig 3:**
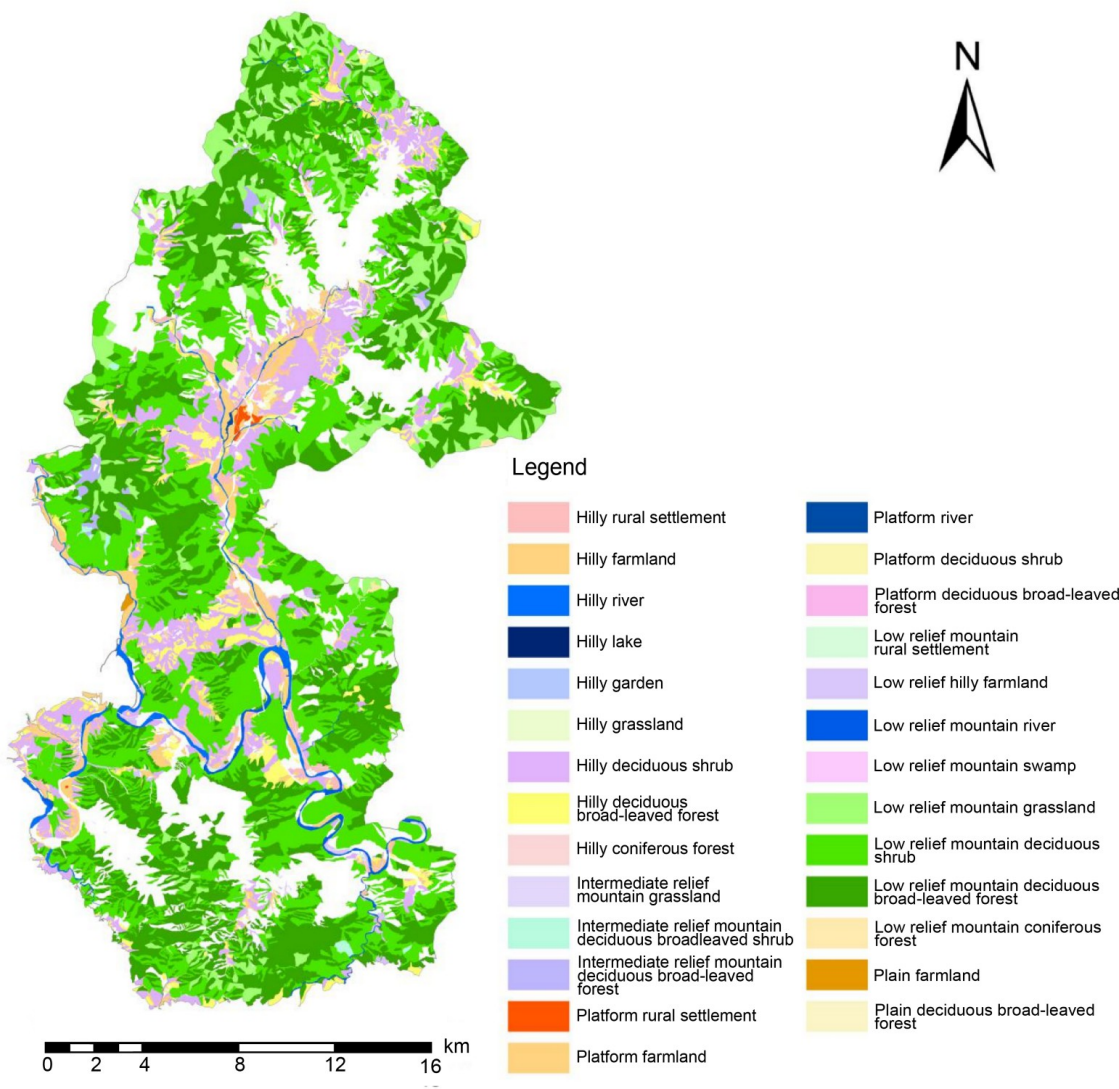
Distribution map of scenic resource groups in non-canyon/river valley areas. (Republished from [https://directory.eoportal.org/web/eoportal/satellite-missions/g/gaofen-2] under a CC BY license, with permission from [Hebei Zhongke Sino Star Information Technology Co., Ltd], original copyright [2018]).

**Fig 4:**
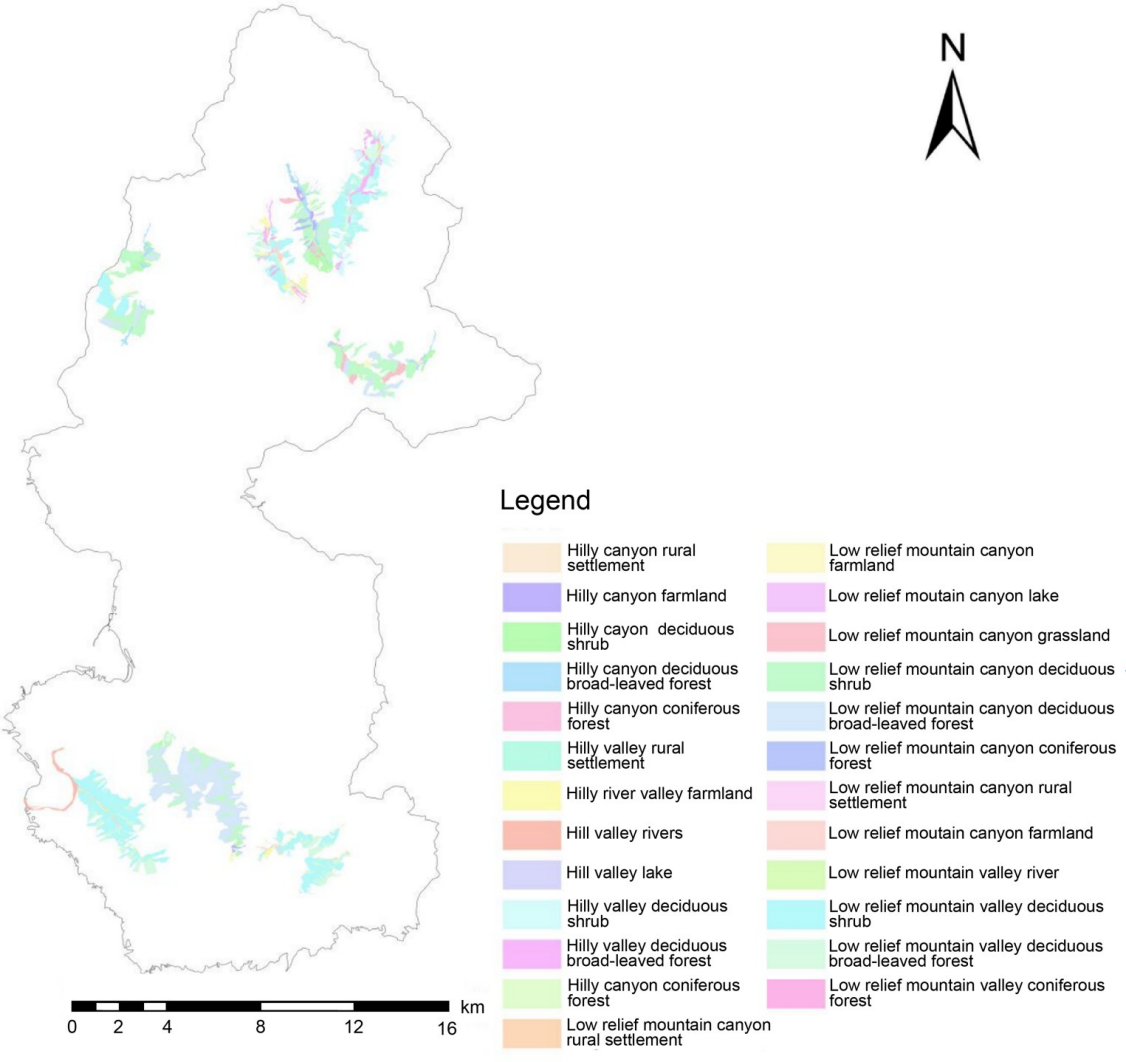
Distribution map of scenic resource groups in canyon/river valley areas. (Republished from [https://directory.eoportal.org/web/eoportal/satellite-missions/g/gaofen-2] under a CC BY license, with permission from [Hebei Zhongke Sino Star Information Technology Co., Ltd], original copyright [2018]).

**Fig 5:**
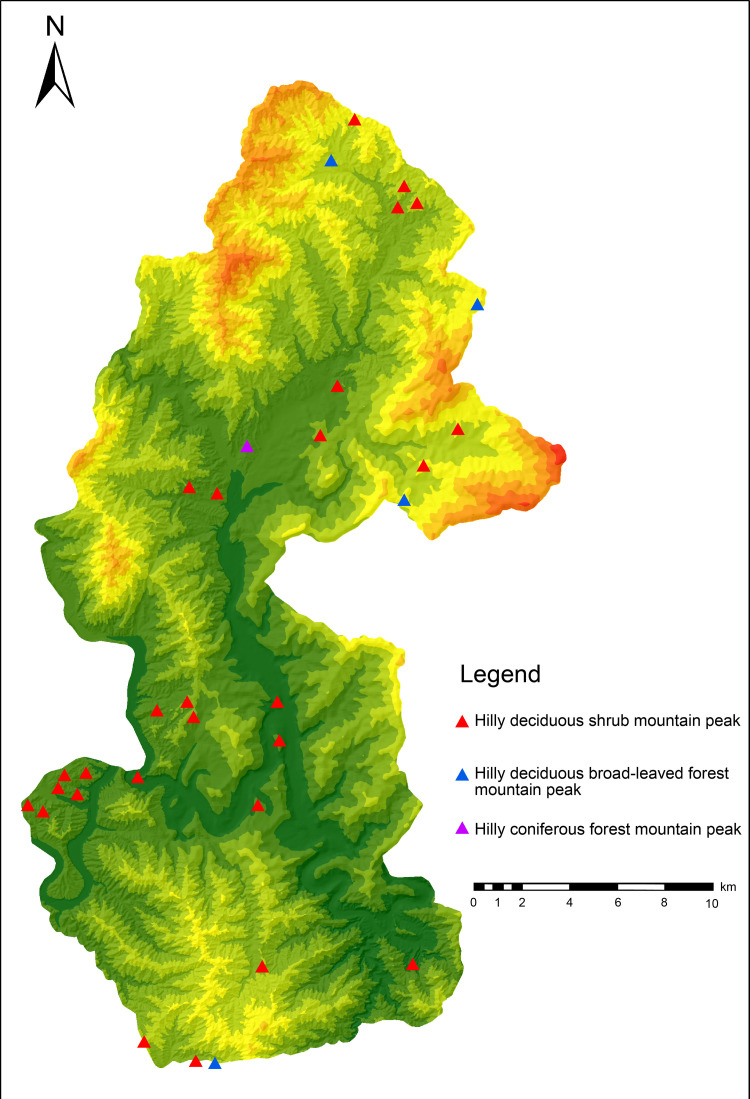
Distribution map of peaks in the hilly area of the Yesanpo National Park. (Republished from [https://directory.eoportal.org/web/eoportal/satellite-missions/g/gaofen-2] under a CC BY license, with permission from [Hebei Zhongke Sino Star Information Technology Co., Ltd], original copyright [2018]).

**Fig 6:**
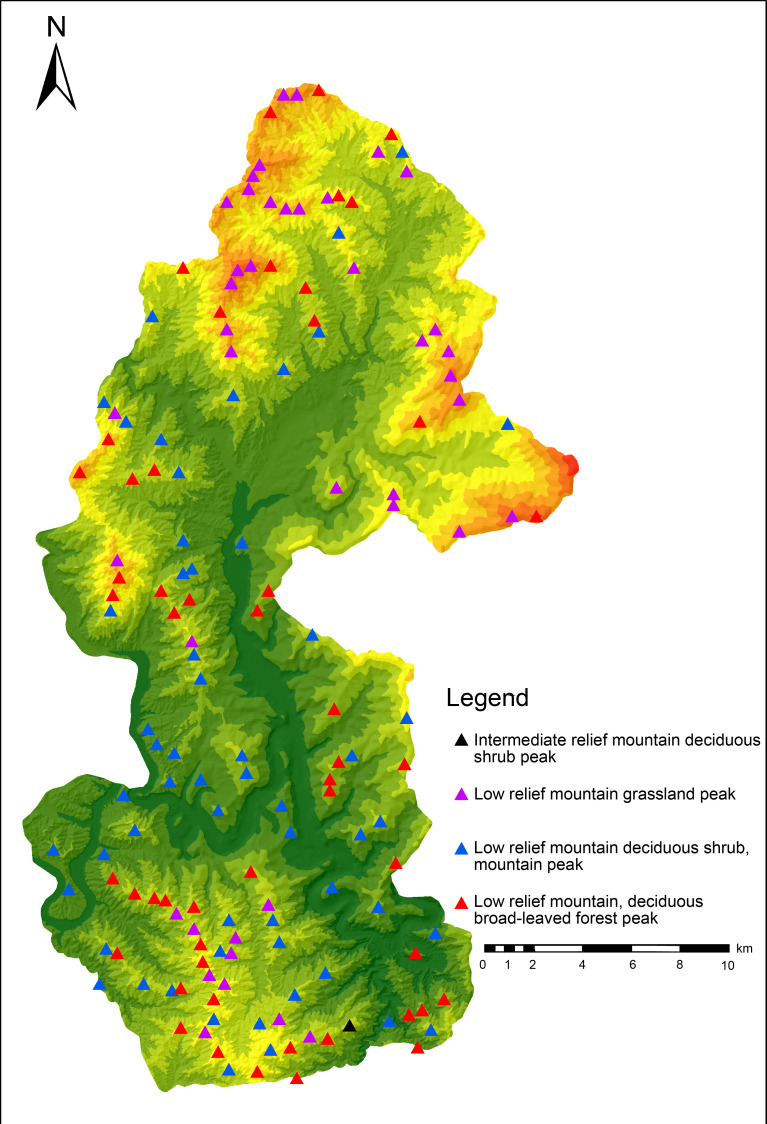
Distribution map of peaks in mountainous area of the Yesanpo National Park. (Republished from [https://directory.eoportal.org/web/eoportal/satellite-missions/g/gaofen-2] under a CC BY license, with permission from [Hebei Zhongke Sino Star Information Technology Co., Ltd], original copyright [2018]).

To efficiently analyze the spatial combination of scenic resources from the perspective of 3D space and identify the scenic resource data of Yesanpo GF-2 images, the object-oriented method was used combined with DEM data, Survey data of forest resource planning and design. ArcScene was used to build a 3D terrain model of the national park [42]. The model contained geographical spatial coordinates, as well as geographical spatial and attribute information [43], as shown in Fig 2. Scenic resource classification data were superimposed to construct scenic resource groups. In this study, the scenic resource groups are polygons and displayed as graphic blocks in GF-2 images, including both developed and undeveloped scenic resources. It was found there were 59 scenic resource groups in the Yesanpo National Park, as shown in Tables 4–6, including 27 types of scenic resource groups in non-canyon/river valley areas, 25 types in canyon/river valley areas, as shown in Figs 3 and 4, and seven types in peaks, as shown in Figs 5 and 6.

**Table 4:** Scenic resource groups in plain and platform areas in non-canyon/river valley area.

No	Scenic resource groups	Area ratio (%)
1	Plain Deciduous Broad-leaved Forest	0.002
2	Plain Farmland	0.043
3	Platform River	0.050
4	Platform Deciduous Shrub	0.082
5	Platform Deciduous Broad-leaved Forest	0.082
6	Platform Farmland	0.474
7	Platform Rural Settlement	0.130
8	Hilly Grassland	0.229
9	Hilly River	1.203
10	Hilly Lake	0.006
11	Hilly Deciduous Shrub	10.451
12	Hilly Deciduous Broad-leaved Forest	4.106
13	Hilly Garden	0.004
14	Hilly Farmland	3.350
15	Hilly Rural Settlement	1.572
16	Hilly Coniferous Forest	0.249
17	Low-relief Mountain Grassland	8.237
18	Low-relief Mountain River	0.731
19	Low-relief Mountain Deciduous Shrub	35.362
20	Low-relief Mountain Deciduous Broad-leaved Forest	31.282
21	Low-relief Mountain + Farmland	0.927
22	Low-relief Mountain Swamp	0.005
23	Low-relief Mountain Rural Settlement	0.139
24	Low-relief Mountain Coniferous Forest	0.554
25	Intermediate-relief Mountain Grassland	0.039
26	Intermediate-relief Mountain Deciduous Shrub	0.194
27	Intermediate-relief Mountain Deciduous Broad-leaved Forest	0.498
	Total	100

**Table 5:** Scenic resource groups in canyon/river valley of the hilly area.

No	Scenic resource groups	Area ratio (%)
1	Hilly Valley River	1.712
2	Hilly Valley Lake	0.193
3	Hilly Valley Deciduous Shrub	3.632
4	Hilly Valley Deciduous Broad-leaved Forest	2.643
5	Hilly Valley Farmland	2.053
6	Hilly Valley Rural Settlement	0.768
7	Hilly Valley Coniferous Forest	0.120
8	Hilly Canyon Deciduous Shrub	2.921
9	Hilly Canyon Deciduous Broad-leaved Forest	2.663
10	Hilly Canyon Farmland	0.656
11	Hilly Canyon Rural Settlement	0.276
12	Hilly Canyon Coniferous Forest	0.255
13	Low-relief Mountain Valley River	0.984
14	Low-relief Mountain Valley Deciduous Shrub	27.621
15	Low-relief Mountain Valley Deciduous Broad-leaved Forest	7.860
16	Low-relief Mountain Valley Farmland	1.126
17	Low-relief Mountain Valley Rural Settlement	0.021
18	Low-relief Mountain Valley Coniferous Forest	0.246
19	Low-relief Mountain Canyon Grassland	1.544
20	Low-relief Mountain Canyon Lake	0.048
21	Low-relief Mountain Canyon Deciduous Shrub	20.255
22	Low-relief Mountain Canyon Deciduous Broad-leaved Forest	21.305
23	Low-relief Mountain Canyon Farmland	0.502
24	Low-relief Mountain Canyon Rural Settlement	0.053
25	Low-relief Mountain Canyon Coniferous Forest	0.543
	Total	100

**Table 6:** Scenic resource groups of peaks.

No	Scenic resource groups	Number	Percentage (%)
1	Hilly Deciduous Shrub Mountain Peak	28	14.21
2	Hilly Deciduous Broad-leaved Forest Mountain Peak	4	2.03
3	Hilly Coniferous Forest Mountain Peak	1	0.51
4	Low-relief Mountain Grassland Peak	42	21.32
5	Low-relief Mountain Deciduous Shrub Peak	67	34.01
6	Low-relief Mountain Deciduous Broad-leaved Forest Peak	54	27.41
7	Intermediate-relief Mountain Deciduous Shrub Peak	1	0.51
	Total	197	100

The indices in Table 2 were used to calculate the spatial distribution type (NNI) and the spatial clustering area of scenic spots (KDE) and analyze the equilibrium degree (Imbalance index) and the concentration degree of scenic resource groups (Geographic concentration index) in the Yesanpo National Park. The results are shown in Table 7.

**Table 7:** Spatial distribution evaluation results.

Spatial distribution evaluation index	Calculation results	Description
NNI	*r*_*1*_ = 510.0697 m for the 135 scenic spots in the Yesanpo National Park, the theoretical nearest distance is *r*_*E*_ = 1017.4398 m, *R* = 0.5013 < 1, *Z*-value is -11.0844, and *P*-value is 0.0001	The type of spatial distribution of scenic spots in the Yesanpo National Park is a clustered type.
KDE	The density of scenic spots in the middle of the Baili Gorge scenery area exceeded 17 scenic spots/km^2^, the density of scenic spots west of the Longmen Tianguan scenery area exceeded 12 scenic spots/km^2^, and the density of scenic spots in the southeast of the Baicao Pan scenery area exceeded 8 scenic spots/km^2^	Spatial distribution of scenic spots in the Yesanpo National Park is notably between regions within the park. The distribution of scenic spots in Baili Gorge scenery area is relatively compact, which is evidently different from that of Jinhua Mountain scenery area in the north, showing a distribution pattern of “dense in the south and sparse in the north.” Three evident spatial distribution clusters have been formed; in descending order of concentration, they are the middle of Baili Gorge scenery area, the west of Longmen Tianguan scenery area, and the southeast of Baicao Pan scenery area, as shown in S1 Appendix. (Republished from [https://directory.eoportal.org/web/eoportal/satellite-missions/g/gaofen-2] under a CC BY license, with permission from [Hebei Zhongke Sino Star Information Technology Co., Ltd], original copyright [2018]).
Imbalance Index	The total number of types of scenic resource groups in the Yesanpo National Park was 59, *S* = 0.8471<1	The spatial distribution of the scenic resource groups in the Yesanpo National Park was extremely imbalanced.
Geographic Concentration Index	*T* = 9528, *n* = 59. Assuming that all types of scenic resources in the Yesanpo National Park were evenly distributed, the average number of spots for each scenic resource was 161.49, Ḡ = 13.02, *G* = 43.10.	The result shows *G>Ḡ*. The difference between the values of G and Ḡ is large. It shows that the spatial distribution of scenic resource groups in Yesanpo National Park is highly concentrated.

### SCISR

The SCISR was used to quantitatively analyze the scenic resource space combination. The expert scoring method was used to score the spatial combination value of scenic resources; 20 questionnaires were issued. The corresponding scores of each scenic resource and its combination type were determined through two rounds of surveys, and the scores were recorded in the scenic resource GIS database (Table 8). The SCISR was calculated to create a SCISR map (S2 Appendix) (Republished from [https://directory.eoportal.org/web/eoportal/satellite-missions/g/gaofen-2] under a CC BY license, with permission from [Hebei Zhongke Sino Star Information Technology Co., Ltd], original copyright [2018]). Through quantitative and graphical analysis, we found that Haitang Valley, Shixuan Gorge, Xiezi Ditch, the west, east, and surrounding areas of the Juma River Basin in the Baili Gorge area, the northeast and western Dalongmen Castle, cliff carvings, and waterfalls in the Longmen Tianguan and surrounding areas, Daze Reservoir, Beiyu Tower, Nanbian Bridge and surrounding areas in Baicao Pan, and the four regions in the middle and southwest of the Jinhua Mountain area showed a higher SCISR (SCISR ≥ 10), and the viewing or utilization value of scenic resource groups was higher, along with the developmental value.

**Table 8:** Scoring table of experts on the value of scenic resources.

Geomorphological layer	Value	Surface cover layer	Value
Plain	1	River	4
Platform	2	Lake	5
Hill	3	Swamp	3
Low-relief mountain	4	Grassland	2
Intermediate-relief mountain	5	Shrub	1
Canyon/river valley	3	Deciduous broad-leaved forest	4
Peak	3	Coniferous forest	4
		Farmland	2
		Rural settlement	1
		Garden	2

**Fig 7:**
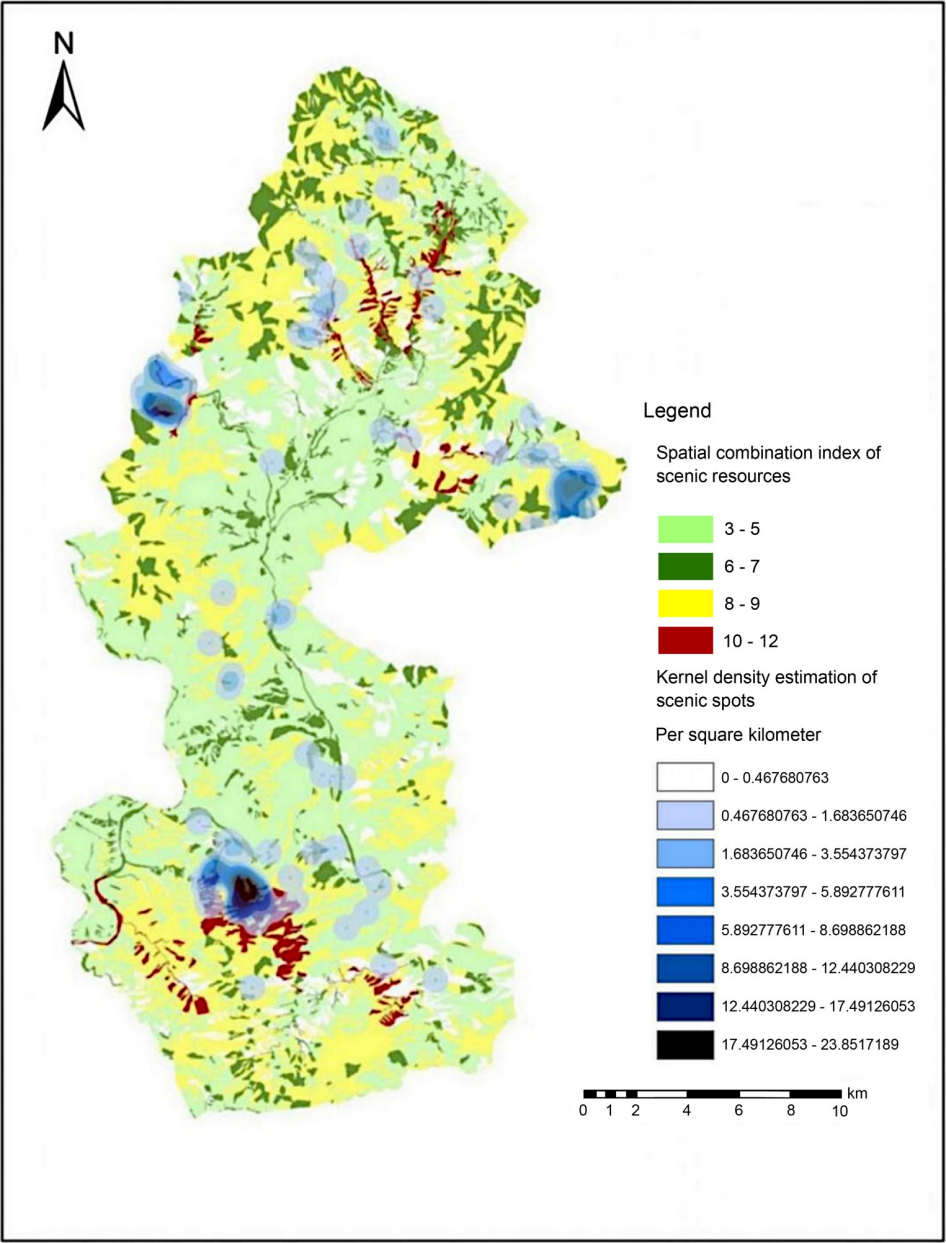
Overlay analysis chart of SCISR and KDE of scenic resources in the Yesanpo National Park. (Republished from [https://directory.eoportal.org/web/eoportal/satellite-missions/g/gaofen-2] under a CC BY license, with permission from [Hebei Zhongke Sino Star Information Technology Co., Ltd], original copyright [2018]).

The KDE map of existing scenic spots was superimposed on the SCISR map, and the pattern between existing scenic spots and the spatial combination of scenic resources was analyzed. Look for areas with a low degree of development, but with a high value for the spatial combination of scenic resources. The results showed there are currently fewer developed scenic spots in the Xiezi Ditch and Juma River basins in the Baili Gorge area, northeast of the Longmen Tianguan area, middle of the Baicao Pan area, and central and southwestern regions of the Jinhua Mountain area. The developmental degree was low, but the SCISR in these areas was relatively high, and the spatial combination value of the scenic resources was also relatively high, which showed high developmental value and great potential, as shown in Fig 7.

## Discussion

According to the spatial distribution of scenic resources, scientifically determining the sequence and measures of its protection and development is the basis for sustainable development of national parks, and is important for integrating and using regional scenic resources, enhancing the overall attractiveness of regional tourism. Evenly distributed scenic resources reflect social equity in tourism development, ecological protection, sustainable development, whereas clustered scenic resources are more conducive to the development of scenic resource groups and connecting tourist routes. However, excessive clustering will inhibit the development of the same type of scenic resources [8], and because the attractions are too concentrated, the pressure on transportation, accommodation, catering, and other facilities will be great. The most suitable travel time for Yesanpo is from April to October. Tourists are concentrated during holidays. For example, from May 1st to 5th, 2021, 350,000 tourists were received, with an average of 70,000 people per day, far exceeding the environmental carrying capacity of the national park, i.e., 60,000 person-times/day [44]. Tourists were mainly concentrated in the Baili Gorge scenery area. The tourist overload in the national park caused traffic congestion, and the imbalance of supply and demand led to problems such as a decline in the quality of tourism services. Overloaded reception and operation accelerate the loss of resources and environmental damage, and seriously damage the protection and sustainable use of scenic resources.

We analyze scenic resources from the perspective of three-dimensional space, determine the spatial distribution types of scenic spots and their spatial clustering areas, determine the balance and concentration of scenic resource groups in spatial distribution, and use the SCISR to quantitatively evaluate the value of the spatial distribution combination of scenic resource groups. The spatial evaluation system of scenic resources constructed in this research can be used to guide national parks to expand tourism space, and integrate internal scenic resources to optimize the layout of tourism space [45]. It provides a scientific basis for scientifically controlling and planning the combination of regional scenic resources and tourism products, optimizing tourism space, creating an all-area tourism-based development model, providing tourists with more high-quality and different types of tourism products. Reasonably diverting tourists and controlling the number of tourists to within the environmental carrying capacity will help solve problems, such as seasonal tourism and traffic congestion, and realize the requirements of obtaining the best economic benefits under the conditions of protecting the quality of scenic resources and not degrading the ecological environment. This will achieve the overall sustainable development of the social, economic, and ecological benefits of the tourism industry in the region [14].

We overlaid the SCISR on the existing scenic spot data to produce the SCISR maps of six scenery areas, evaluated the scenic resources of each scenery area in the Yesanpo National Park from the perspective of the SCISR, and analyzed the existing problems of scenic resources, finally providing ideas for the sustainable development of each scenery area.

### Baili Gorge area

The area is located in the southwest of the Yesanpo National Park. The surrounding SCISR in the central part of the area, the west and east of the Juma River basin, is 10–12, which indicates a higher SCISR. It is the area with the most abundant and rich scenic resources and the best combination in the national park, such that its viewing and utilization value is high. The area is characterized by several resources, such as canyons, mountains, water sources, forests, deep valleys, peaks, forests, cliffs, streams, and waterfalls. The typical scenic spots are the surroundings of the Haitang Valley, the Shixuan Gorge, and the Xiezi Ditch. There are rich scenic resources, a good combination of views with a high developmental value, and high development potential. This is the core area of Yesanpo.

The main existing problems are as follows:

1. Because the main scenic resources are located in the canyon, the scope of viewing and recreation is small, and during the peak period of the tour, it is easy to cause congestion.
2. The scenic resources in the Xiezi Ditch, the west and east of Juma River basins and the surrounding areas are concentrated and the developmental value is high; therefore, they must be reasonably developed.

Some development ideas include the following:

Theme of the area: Strange gorge, deep valley, geological wonders.

1. Protect scenic resources and highlight the characteristics of a canyon. The area is characterized by canyons, mountains, water, forests, and other landscapes. The canyon has a peculiar structure, which belongs to the typical landform of a narrow gorge. It is rich in scenic resources in the canyon area and has high developmental value. It is suitable for sightseeing, mountaineering, scientific examination, exploration, leisure, and vacation. The next step should strengthen the protection of existing scenic resources and show each scenic resource one by one, expressing its broad landscape.
2. Scientifically development of the area with a better combination of scenic resources to enhance tourist experience. To date, the development of the area is concentrated mainly around Shixuan Gorge and Haitang Valley, which is the most concentrated area for tourists in the Yesanpo National Park. During the peak period of the tour, congestion likely affects the tour experience, and the area is prone to danger. The next step is to develop Xiezi Ditch, which has a good combination of scenic resources, while extending the tour area to the south and east, developing the southeast mountain area, increasing mountaineering, scientific research, leisure, and other projects, and improving the utilization value of the combination of scenic resources.
3. Pay equal attention to the development and protection of scenic resources. To promote the sustainable utilization of scenic resources, the management department of the scenery area should start an ecological restoration project to renovate the area and stop the destruction of mountains and vegetation caused by constructing roads and disorderly development.

### Baicao Pan area

The area is located in the northeast of the Yesanpo National Park. The SCISR of Daze Reservoir, Beiyu Tower, Nanbian Bridge, and the surrounding scenic resources is 10–12. This is an area with a higher SCISR, with rich scenic resources and a better combination of views. Its viewing and utilization value is high. It is mainly characterized by high mountains and plant resources. The scenic resources are mainly concentrated in the middle altitude in the east, the ecological environment is relatively fragile, the developmental value is high and the development potential is large. This is the core scenery area of Yesanpo.

The main existing problems are as follows:

1. The Daze Reservoir, Beiyu Tower, Nanbian Bridge, and surrounding scenic resources in the central part of the area are relatively concentrated, with high developmental value, which require reasonable development.
2. The types of scenic resources are single and seasonal.
3. There is a single route to and from the area, which is prone to congestion during peak seasons.
4. There is a lack of links to other areas.

Some development ideas include the following:

Theme of the area: Forest meadow.

1. Protect forest vegetation resources and highlight natural scenery. The main peak of the area is at the top of the monoclinic mountain. This is the highest peak in the Yesanpo National Park. It stands side by side with the neighboring sister peak, Baihua Mountain (1991 m) in Beijing, the highest peak in the west of Beijing. The natural vertical band spectrum of vegetation is evident and rich. The vegetation landscape of the original forest and the original secondary forest is apparent. The next step should be to strengthen the protection of existing scenic resources, especially forest vegetation resources, highlighting birch forests, rhododendrons, Syringa reticulata, alpine meadows, and other plant landscapes, embody the distinctive characteristics of the four seasons, and gradually develop Daze Reservoir, Beiyu Tower, and Nanbian Bridge area.
2. Improve infrastructure and link surrounding areas. Improve the scenic tour system and upgrade service facilities. Strengthen the linkage with other scenery areas of Yesanpo, Beijing Baihua Mountain Scenic Area, and Shangfangshan National Forest Park by increasing comprehensive slow-moving roads and public transportation, promoting the regional linkage development of the Yesanpo National Park.

### Longmen Tianguan area

This area is located northwest of the Yesanpo National Park. The SCISR is 10–12 in the northeast, western Dalongmen Castle, cliff rock carvings, waterfalls, and the surroundings, which belong to an area with a high SCISR. It is an area with a good combination of scenic resources, featuring high mountain waterfalls, strange rocks, canyons, and forest landscapes, rich in scenic resources, with high viewing, utilization and developmental value, and great development potential.

The main existing problems are as follows:

1. The scenic resources in the northeast of the area are relatively concentrated and the developmental value is high; the resources need sustainable development.
2. Natural resources, especially the combination of high mountains and waterfalls, are fragile and vulnerable to damage.
3. The cultural image of Great Wall is not clear enough and publicity is insufficient; it has not yet become the core tourist attraction of the Yesanpo National Park.

The following are some development ideas:

Theme of the area: Tianguan waterfall, cultural corridor.

1. Protect scenic resources and create natural scenery features. Protection of scenic resources in the Caishu’an area and surrounding touristic areas. Appropriate expansion of the scope of the core tourist area, better excavation and presentation of the unique natural landscape, and use of the rich natural scenic resources, such as waterfalls, strange rocks, canyons, and forests. Advantages include highlighting natural scenery with high mountains and waterfalls as the core.
2. Highlight wild interest and rationally develop river scenery resources. With the characteristics of the nature and wild, the Xiaoxi River should be reasonably developed, and the protection of water resources should also be considered during development.
3. Relying on the culture of the Great Wall to create high-quality humanistic scenic resources. Strengthening the protection of key cultural relics of the Dalongmen Ancient Castle, conducting more in-depth and textual research, exploring the culture of the Great Wall, and developing cultural tourism and research travel markets.
4. Scientifically setting up tour routes to organically connect natural and cultural scenic resources. The area has many types of natural and human scenic resources, a high degree of combination, a concentrated spatial distribution, and a high developmental value. Through a reasonable setting of tourist routes, the western Dalongmen Castle, cliff carvings, and high mountain waterfalls can be organically connected with the northeast scenic resources to improve the quality of the scenery area and build it into one of the main scenery areas in Yesanpo.

### Jinhua Mountain area

The area is located in the northern part of the Yesanpo National Park. The SCISR of Qingquan Temple, Zhuangli Reservoir, Cypress House, and surrounding scenic resources in the middle and southwest of the scenery area is 10–12, which indicates an area with a higher SCISR, featuring mountains, waterfalls, and forests, with high viewing, utilization, and developmental value.

The following are the main existing problems:

1. The scenic resources in the central and southwestern parts of the area are relatively concentrated, and the developmental value is high; they need to be reasonably developed.
2. Insufficient protection of cultural resources in villages.
3. The overall development level is not high, the tourist service facilities are obviously insufficient, and there is a lack of connection between scenic resources.

Some development ideas include the following:

Theme of the area: Rolling peaks and valleys, Xanadu.

1. Priority protection and rational development of scenic resources. Most of the area to the west of the Provincial Highway S236 belongs to the Jinhua Mountain-Henglingzi Brown-Eared Pheasant Nature Reserve. Ecological protection and cultivation should be prioritized here to reduce the negative impact of human activities on scenic resources and make this area a natural green barrier for the entire area. The Zhuangli Reservoir and surrounding scenic resources in the central part of the scenery area are relatively concentrated, with high developmental value, and can gradually be developed for leisure, sports, water tourism, and other projects.
2. Protect humanistic scenic resources and explore traditional culture. Strengthen the protection and development of the Qingquan Temple in the southwest and the ancient residential buildings of Lingnantai in the north. Excavate the culture and folklore of Sanpo mountain dwellings, and achieve rural tourism.
3. Connecting greenways to strengthen the link between scenery areas. Scenic resources are further developed in the central and southwestern parts of the area, connecting the Zhuangli Reservoir, Qingquan Temple, and Lingnantai ancient residential buildings through highways and comprehensive slow-moving roads, as well as the Longmen Tianguan scenery area in the southwest with the corresponding service facilities, which form a special tour with a leisure theme, water sports, and traditional culture experience.

### Juma River area

The area is located in the southeast of the Yesanpo National Park. The SCISR of the scenic resource groups in the scenery area are 3–5, indicating a low value for viewing or use. The central, southern, and eastern combination indices are relatively high, with the Juma River and its tributaries as the core landscape, the type of scenic resources is single, and the river and its shores have a certain developmental value.

The main existing problems are as follows:

1. Scenic resources are mainly single type rivers, lacking representative landscapes with a single-tour structure.
2. Some buildings along the river are inadequately set back and are located within the planned river regulation line, which affects the waterfront landscape.
3. There are few types of plants and a lack of characteristic views.

Some development ideas include the following:

Theme of the area: Green hill, green shadow, Water Rhyme Gallery.

1. Renovate the river and coastal environment to build the Beijing West Gallery. The area stretches along the Juma River and its tributary Xiaoxi River. The riverside is an important external traffic source that passes through the entire area, and most towns, villages, and other residential areas are concentrated along the river and the road. Therefore, the focus of the construction was to create landscapes along the river. Protecting the ecological environment of the mountains and forests along the river should be strengthened, the landscape should be improved, and the damaged landscape should be ecologically restored. By strictly adhering to the “Hebei Province Blue Line Management Regulations” within the river regulation line and prohibiting illegal construction, the river bank should maintain its ecological and natural nature. Simultaneously, wild flowers and color-leaf plants that reflect local characteristics can be used to create a botanical landscape with four seasons, colorful, and local characteristics.
2. Build riverside greenways to meet leisure needs. A slow-moving greenway along the riverside can be constructed along the entire Juma River, connecting various scenery areas, creating an aesthetic and leisure road.
3. Relying on scenic resources and facilities along the road, water tourism, strolling, cycling, and other excursions can be conducted.

### Yugu Cave area

The area is located in the middle of Yesanpo National Park. Most areas in the scenery area have an SCISR of 3–5, indicating a low SCISR, but the viewing and utilization value of the scenery area is not low. The reason for this phenomenon is that the most distinctive Yugu Cave and Yugu Spring are karst caves and spring scenery resources in the scenery area. They are located underground and cannot be identified using RS images and DEM data. Therefore, the score of the SCISR is low.

The following are the main existing problems:

1. The types of scenic resources are single and there is a lack of connection among various scenic resources.
2. The protection and utilization of war relics, such as egg tuo and the historical relics of the Buddha Cave Tower and other scenic resources are insufficient and they have not been included in tourist attractions.
3. Damage to the mountain caused by road construction is evident, and ecological restoration is urgently needed.

Some development ideas include the following:

Theme of the area: Enjoyable cave, clear pond, and unique spring.

1. Strengthen the protection of Yugu Cave and Yugu Spring, highlighting the characteristics of “spring” and “cavity.” Increase the protection of Yugu Cave and Yugu Spring, conduct scientific investigations, highlighting their cultural connotations and scientific value.
2. Exploit humanistic scenic resources and highlight the local culture. The battle site of the Pingxi Base Area was excavated based on the deeds of the five warriors of the egg tuo.

According to the comprehensive evaluation results (Fig 7), the spatial distribution of scenic resources in Yesanpo National Park was relatively low in balance, and the distribution was relatively concentrated, exhibiting a clustered spatial distribution pattern. The area with a higher SCISR partly follows the existing planning and development hotspots of the National Park, the areas with high density of scenic spots (the middle of the Baili Gorge, west of the Longmen Tianguan, and southeast Baicao Pan areas). Besides the existing planning and development hotspots, the west, east, and surrounding areas of the Xiezi Ditch, Juma River basins in the Baili Gorge area; the northeast of Longmen Tianguan area, Daze Reservoir, Beiyu tower, Nanbian bridge, and surrounding areas in the central Baicao Pan area; and the central and southwest areas of the Jinhua Mountain area have less developed scenic spots and a low developmental degree, which attract the attention of planning and management departments. The SCISR in these areas was high with prominent developmental value. Therefore, these areas need scientific planning and management to promote the sustainable development of the national park.

By summarizing the related studies on the evaluation of scenic resources in the Yesanpo National Park, it is found there are few related documents—less than 10 papers. Furthermore, most previous studies used data collection and sorting, the field survey method [46], questionnaire survey method [47], mathematical statistics methods (analytic hierarchy process [48], fuzzy evaluation method [49, 50]), comprehensive analysis method (SWOT Analysis [44]), and other social research methods; only one study used GIS to evaluate the scenic resources of Yesanpo. In these studies, Xie, Sun, Liu, and Cui focused on the macro-evaluation of the scenic resources of the entire Yesanpo National Park. The results showed Yesanpo is rich in scenic resources, especially natural scenic resources, but the current development is imbalanced and has high developmental value. Liang used a questionnaire survey, the Delphi method, and analytic hierarchy process to evaluate tourism resources. High-quality tourism resources are concentrated in Baili Gorge, Longmen Tianguan, and Yugu Cave scenery areas, which are also the focus of development and protection [47]. Li Xiaofeng used the analytic hierarchy process and multi-level comprehensive fuzzy evaluation method to evaluate the development potential of the Baili Gorge, Longmen Tianguan, and Yugu Cave scenery areas. The results show that among the three scenery areas, the Baili Gorge area has the best development potential, the Longmen Tianguan area has the second best development potential, and the Yugu Cave area has the lowest development potential [50]. Through GIS overlay and buffer analysis, Sun Qiuhua showed that the geological relics of the Baili Gorge and Longmen Tianguan have “linear” and “circular” distribution characteristics [25]. The research results of this study are consistent with those of previous studies. Previous research focuses more on the macro-evaluation of the scenic resources of the entire scenic area. It is inaccurate enough and the evaluation objects are not specific to the scenic spots, which can support for the tourism development strategy. This study evaluates scenic resources from a three-dimensional space, and the results obtained are more objective and specific. Besides providing support for tourism development strategies, it can provide more scientific and precise scientific support for tourism planning and sustainable development.

Therefore, we proposed a comprehensive spatial identification and evaluation system for scenic resources based on 3S technology. This study used multisource data (GF-2 imagery, DEM, forest resource planning, and design survey data) and multiple platforms (ArcGIS, ENVI, eCognition) to identify and evaluate scenic resources. Based on the evaluation model of the evaluation criteria, such as the NNI, KDE, imbalance index and geographic concentration index models, and the spatial characteristics of scenic resources, an SCISR evaluation model is proposed. The SCISR is regionally more specific than the previous landscape or geographic evaluation standards. It can graphically present the spatial combination evaluation results, and the results obtained are more objective and accurate. After verifying the results of the Yesanpo National Park field survey as well as previous studies, it was found that the identification and evaluation results of this method better reflect the actual situation of the scenic resources, have high consistency, and prove the feasibility and reliability of the method. They can provide a scientific reference basis for the sustainable development and utilization of scenic resources and related planning work.

We have explored and improved the field of research on the spatial distribution of scenic resources, and our innovations are reflected in the following three points:

1. In terms of research theory, previous studies were mainly based on theories of geography [11–13]. This study combines geography, forestry, ecology, tourism, geomorphology, and landscape architecture to promote the integration of multiple disciplines, and uses multidisciplinary theories and technologies to solve practical problems in the evaluation of scenic resources in national parks.
2. In terms of research objects, previous studies mostly focused on the planar spatial structure of tourist cities and tourist attractions [14]. According to the composition structure of the scenic resources on the surface, this research studies the distribution characteristics of the scenic resources in a three-dimensional space. Compared to a two-dimensional plane, it can better reflect the actual characteristics of scenic resources, and the data obtained are more scientific and objective.
3. In terms of research content and methods, previous studies have mainly focused on a single platform and a single data source to evaluate the planar spatial structure of scenic resources [15–21]. This study uses multiple platforms and multiple data sources to conduct a more comprehensive analysis of developed and undeveloped scenic resources from the perspective of three-dimensional space, determine the spatial distribution types of scenic spots and their spatial aggregation areas, and determine the balance of the scenic resource groups in the spatial distribution. The SCISR is proposed for the first time and it is used to analyze the value of scenic resource spatial combination.

China’s existing national park planning policies do not require the evaluation of the spatial combination of scenic resources. However, as the government and the public are paying increasing attention to the sustainable development of national parks, one of the important tasks for the planning and management departments is the comprehensive and accurate identification and evaluation of scenic resources. The identification and evaluation system proposed herein can provide a scientific basis and an easy-to-implement evaluation system for improving the planning and management of national parks, meeting the actual needs of planning and management departments and rendering them easy to operate and promote. Furthermore, the rapid development of 3S technology has facilitated big data collection and analysis. Using multisource data and multiple platforms for conducting spatial evaluation research of scenic resources is a key topic that requires comprehensive studies, and this study can provide a reference basis for this research field.

This study has primarily three limitations. First, the time dimension was not added while evaluating the spatial distribution of scenic resources. In future research, it is necessary to consider the influence of time factors, such as annual and seasonal changes, on the spatial distribution of scenic resources. Second, the identification and evaluation system needs to be supplemented and improved. The different types of spatial combination and evaluation indicators of the scenic resources need to be further supplemented, refined, and improved. For example, a combination of scenic resources, such as weather landscape, animal resources, and human landscapes, needs to be added. Moreover, comprehensively integrating the results in the fields of landscape architecture, geography, ecology, aesthetics, history, and art can supplement and improve the spatial evaluation indicators of scenic resources. Third, the data sources used in this study can be further expanded and optimized. In the future, we can attempt to introduce technologies such as higher-resolution RS images and drone tilt photography for the identification and evaluation of scenic resources to meet the requirements of national parks with different precision, different planning, and sustainable development management requirements.

## Conclusions

Social and economic developments have increased problems with the sustainable use of scenic resources, which has now become one of the research hotspots in this field. The key is to identify and evaluate the spatial distribution of scenic resources and conduct their scientific planning and management. We used GPS field measurements, RS image recognition, GIS spatial analysis, mathematical methods in geography, based on multiple data sources and platforms, to identify and evaluate scenic resources, which is technically difficult and highly comprehensive. Moreover, SCISR was proposed for the first time to evaluate the value of scenic resources from the perspective of spatial combination. The NNI, KDE, imbalance index, and geographic concentration index models were used as indicators of the spatial distribution characteristics of the scenic resources, which promote the quantitative and graphical evaluation of the spatial distribution of the scenic resources, helping units and related departments to understand the spatial distribution of the scenic resources more comprehensively, identify areas with relatively concentrated scenic resources, high developmental value, and greater development potential, and provide data support for decision making in national park planning and management. On-site verification was consistent with the actual data. Therefore, several indices can be considered important indicators for the evaluation of the spatial distribution of scenic resources and planning of national parks. This study provides the theoretical foundation and technical support for high-precision digital and systematic evaluation of scenic resources.

The multisource data used in this study are multiperiod and digitized data with characteristics such as stability and high accuracy, which can reduce the workload and difficulty of field investigations and improve the efficiency of planning and management of scenic resources. The methodology and system are easy to popularize and apply, and thus, can be incorporated into the national park planning and management system. In addition, based on previous studies, this research has contributed to the identification and evaluation system of the spatial distribution of scenic resources. However, with continuous technological development, future research should attempt to combine scenic resource identification and evaluation systems with advanced technologies, such as big data, artificial intelligence, and three-dimensional virtualization, to improve the automation and intelligence of scenic resource identification and evaluation.

## Supporting information

S1 Appendix (TIF)

S2 Appendix (TIF)
