# Supplementary figures and images for: Evaluation of the spatial distribution of scenic resources based on 3S technology: A case study of the Yesanpo National Park
Source: PLoS One. 2022 Jul 8;17(7):e0269841. doi: 10.1371/journal.pone.0269841 (PMC9269949; doi:10.1371/journal.pone.0269841)

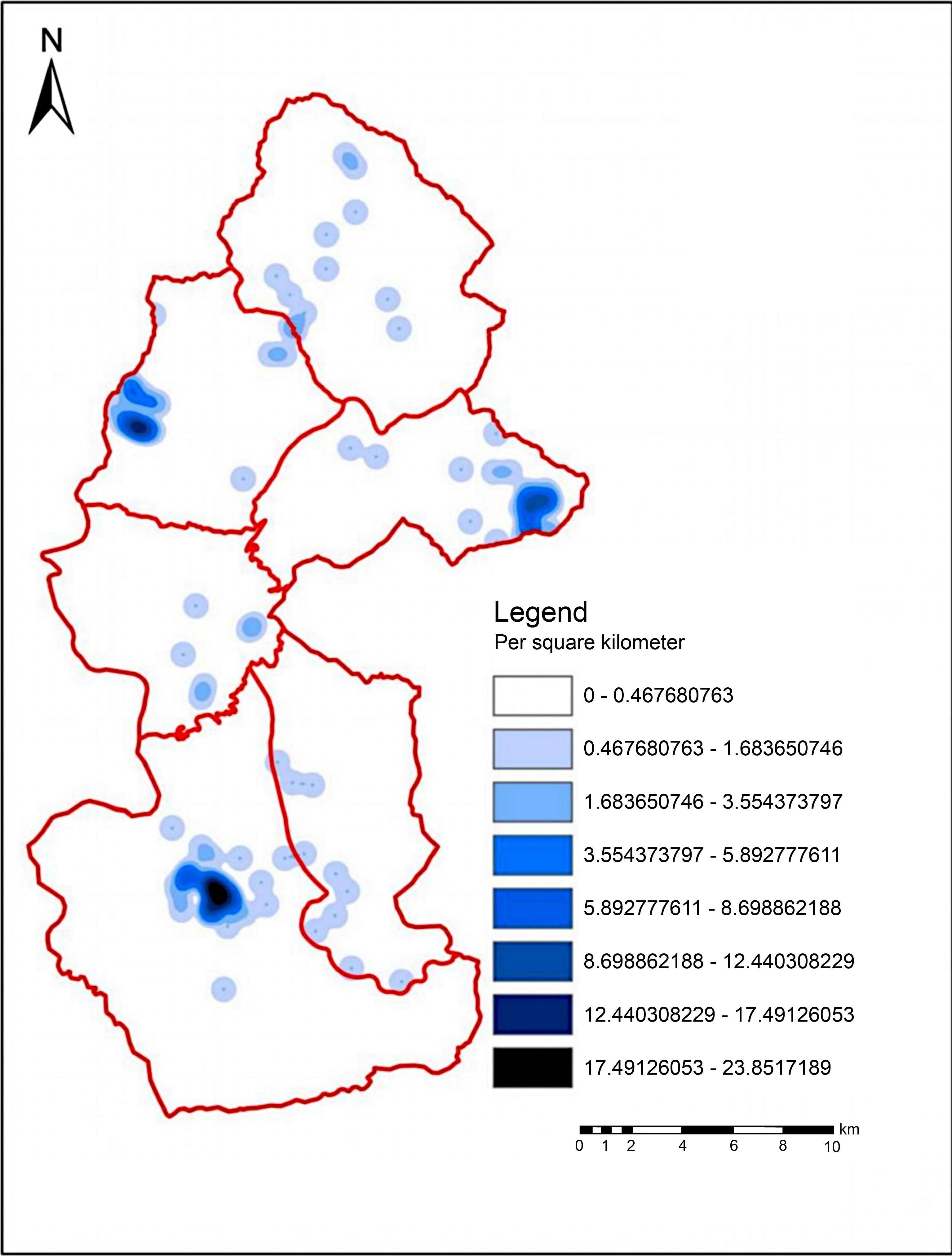

Supplement: S1 Appendix — (TIF) [file pone.0269841.s001.tif]

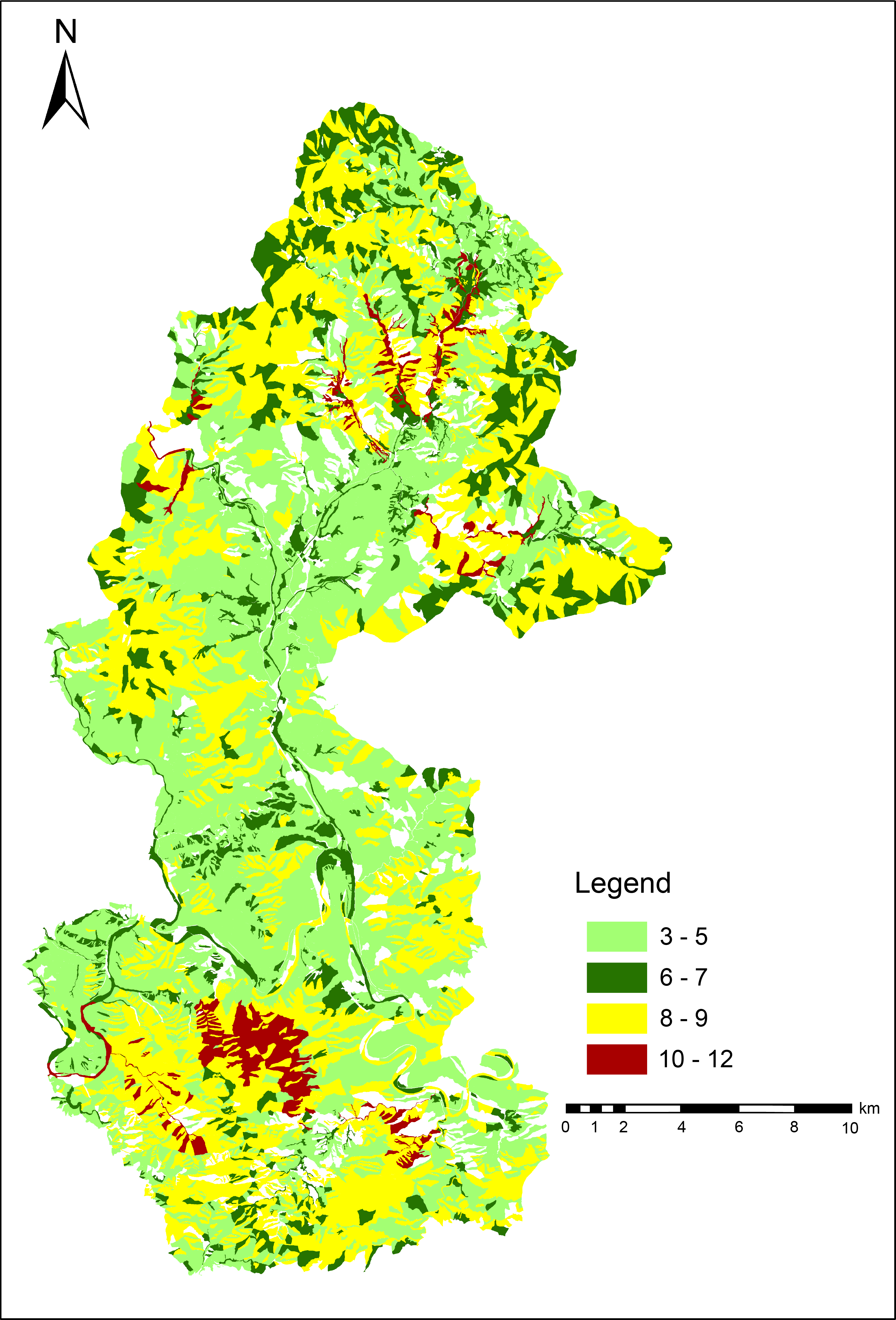

Supplement: S2 Appendix — (TIF) [file pone.0269841.s002.tif]
